# Analysis on Burnout, Job Conditions, Alexithymia, and Other Psychological Symptoms in a Sample of Italian Anesthesiologists and Intensivists, Assessed Just before the COVID-19 Pandemic: An AAROI-EMAC Study

**DOI:** 10.3390/healthcare10081370

**Published:** 2022-07-24

**Authors:** Alessandro Vittori, Franco Marinangeli, Elena Giovanna Bignami, Alessandro Simonini, Alessandro Vergallo, Gilberto Fiore, Emiliano Petrucci, Marco Cascella, Roberto Pedone

**Affiliations:** 1 Department of Anesthesia and Critical Care, ARCO ROMA, Ospedale Pediatrico Bambino Gesù IRCCS, 00165 Rome, Italy; 2 Department of Anesthesiology, Intensive Care and Pain Treatment, University of L’Aquila, 67100 L’Aquila, Italy; francomarinangeli@gmail.com; 3 Simulearn, Simulation Center of AAROI-EMAC, 40121 Bologna, Italy; vergallo@aaroiemac.it; 4 Anesthesiology, Critical Care and Pain Medicine Division, Department of Medicine and Surgery, University of Parma, 43121 Parma, Italy; elenagiovanna.bignami@unipr.it; 5 Pediatric Anesthesia and Intensive Care Unit, Salesi Children’s Hospital, 60121 Ancona, Italy; dr.simonini@gmail.com; 6 Department of Anesthesia and Intensive Care, Spedali Civili di Brescia, 25121 Brescia, Italy; 7 Department of Anesthesia and Intensive Care, Hospital of Santa Croce di Moncalieri, 10024 Turin, Italy; gilberto.fiore@gmail.com; 8 Department of Anesthesia and Intensive Care Unit, San Salvatore Academic Hospital of L’Aquila, 67100 L’Aquila, Italy; petrucciemiliano@gmail.com; 9 Department of Anesthesia and Critical Care, Istituto Nazionale Tumori—IRCCS, Fondazione Pascale, 80131 Naples, Italy; m.cascella@istitutotumori.na.it; 10 Department of Psychology, University of Campania Luigi Vanvitelli, 8100 Caserta, Italy; roberto.pedone@gmail.com

**Keywords:** burnout, anesthesiology, anesthesiologist, intensivist, mental health, alexithymia, health care professionals, anxiety, personal accomplishment, emotional exhaustion

## Abstract

**Background**. It was previously reported that health care professionals working in the fields of anesthesiology and emergency are at higher risk of burnout. However, the correlations between burnout, alexithymia, and other psychological symptoms are poorly investigated. Furthermore, there is a lack of evidence on which risk factors, specific to the work of anesthetists and intensivists, can increase the risk of burnout, and which are useful for developing remedial health policies. **Methods**. This cross-sectional study was conducted in 2020 on a sample of 300 professionals recruited from AAROI-EMAC subscribers in Italy. Data collection instruments were a questionnaire on demographic, education, job characteristics and well-being, the Maslach Burnout Inventory Tool, the Toronto Alexithymia Scale, the Symptom Checklist-90-R, and the Rosenberg Self-Esteem Scale administered during refresher courses in anesthesiology. Correlations between burnout and physical and psychological symptoms were searched. **Results**. With respect to burnout, 29% of individuals scored at high risk on emotional exhaustion, followed by 36% at moderate–high risk. Depersonalization high and moderate–high risk were scored by 18.7% and 34.3% of individuals, respectively. Burnout personal accomplishment was scored by 34.7% of respondents. The highest mean scores of burnout dimensions were related to dissatisfaction with one’s career, conflicting relationships with surgeons, and, finally, difficulty in explaining one’s work to patients. **Conclusions**. Burnout rates in Italian anesthesiologists and intensivists have been worrying since before the COVID-19 pandemic. Anesthesiologists with higher levels of alexithymia are more at risk for burnout. It is therefore necessary to take urgent health policy measures.

## 1. Introduction

According to the 11th Revision of the International Classification of Diseases (ICD-11) of the World Health Organization (WHO), Burnout (BO) is an occupational phenomenon that is defined as a “syndrome conceptualized as resulting from chronic workplace stress that has not been successfully managed” [1]. This syndrome is characterized by three dimensions, including “feelings of energy depletion or exhaustion; increased mental distance from one’s job, or feelings of negativism or cynicism related to one’s job; and reduced professional efficacy” [2]. Of note, among health care professionals (HCPs), BO has a prevalence that, in certain categories and at higher risk of stress, can reach 50% [3,4]. In particular, it was previously reported that about one-half of intensivists presented a high level of BO, and up to 59% of anesthesiologists exhibited some high-risk BO features [5,6].

These important numbers must lead the scientific community to study BO in a more systematic way, to develop, in turn, effective preventive strategies and health policies. It is mandatory to perform a multidisciplinary approach that presupposes close collaboration between clinicians and psychologists who are experts in this matter. BO is a phenomenon that affects HCPs and, more generally, all helping professions. However, this phenomenon must be contextualized, as the same profession exercised in different contexts exposes to different risks of BO [4]. In addition, each health profession has peculiarities (type of study path, occupational risks, remuneration, etc.) exposed to specific risks of BO and specific risk factors. Anesthesiologists and intensivists carry out a job that involves a long course of study, exposure to moments of great tension alternating with moments of waiting, and, above all, the need to make decisions in a short moment [7]. As if this were not enough, the history of anesthesiologists is relatively recent, with a nursing origin and a belated definition of skills and a precise training path [8]. For these reasons, studies have been conducted on the incidence of BO in different countries, as the results can be extremely different from context to context [6,9,10,11,12,13]. However, these studies performed a descriptive analysis of BO incidence among anesthesiologists and their quality of life. Furthermore, the Italian study by Sanfilippo et al. [10] focuses on a particular population of anesthesiologists: cardiac anesthesiologists.

The Maslach Burnout Inventory Tool (MBI) is a commonly adopted instrument for evaluating the BO phenomenon, even in HCPs [14]. In addition to this instrument, several attempts have been made to better characterize the phenomenon. Hobfoll and Shirom, for instance, have defined BO as decreased enthusiasm about work, hopelessness, and feelings of entrapment [15]. Furthermore, numerous factors may affect the development of BO, and these factors mostly include demographic and job characteristics (e.g., skill variety, task significance, autonomy, and feedback from the job). Indeed, BO can be associated with other psychological symptoms. For example, since alexithymia represents a psychological construct characterized by difficulties in processing emotions that interferes with the mechanisms of self-regulation and reorganization of emotions (“no words for feelings”), and anesthesiologists often have to deal with situations with high emotional impact, it would be interesting evaluating the potential association between BO and alexithymia in this setting of HCPs [16].

On these bases, this study was aimed at evaluating the phenomenon of BO among anesthesiologists. In particular, we investigated (a) the occurrence of BO; (b) the relation between employee BO and job satisfaction relatively to demographic, education, and job characteristics; and (c) the relationship between several psychological (e.g., alexithymia) and physical symptoms and demographic, education, and job characteristics.

Therefore our study evaluated the prevalence of BO, stratifying the risk but also analyzing the correlations with alexithymia and other psychological symptoms. Moreover, we investigated the potential causes, specific to anesthesiologists and intensivists, on which it is possible to develop remedial health policies

## 2. Materials and Methods

### 2.1. Participants

**Figure 1:**
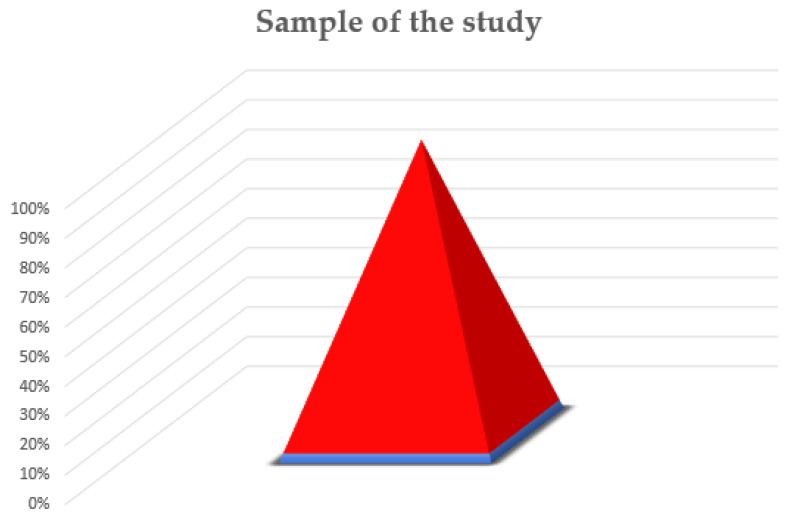
Sample of the study. Legend. Blue: participants of the study; Red: AAROI-EMAC subscribers.

All physicians included in our sample had to be a member of the anesthesia association AAROI-EMAC (Italian Association of Hospital Anesthesiologists, Pain Medicine Specialists, Critical Care and Emergency Physicians), which has around 10,000 subscribers, and actively practicing [17]. The sample was collected via a questionnaire that was administered in the presence of all those who attended refresher courses at the AAROI-EMAC simulation center in February 2020 (Figure 1). Of the 310 respondents, 300 (96.77%) had complete information for the variables used in the current study. All measures were included in a battery of questionnaires as part of a larger-scale data collection project for numerous studies in the fields of psychology, medicine, and clinical risk management.

The survey was composed of 7 sections: (1) Demographic Data, (2) Turnover Intent, (3) Personality, (4) Burnout, (5) Work Engagement, (6) Work Context, and (7) Job Satisfaction.

All subjects volunteered to participate after being presented with a detailed study description, and all were treated in accordance with the “Ethical Principles of Psychologists and Code of Conduct”. The study received the approval of the Ethics Committee of L’Aquila and Teramo, Protocol Number: 0024436/20. We excluded individuals who indicated that they had a history of psychiatric diagnoses and/or substance-related disorders. Those with a history of psychotropic drugs were also excluded (Table 1).

All subjects were Italian citizens who worked in Italy as anesthesiologists; 101 participants (33.67%) were male, and 199 (66.33%) were female. Participants were divided into five age groups: 25–29 years, 30–39 years, 40–49 years, and >49 years. Based on marital status, 81 subjects were unmarried, 196 married, 11 separated, 11 divorced, and 1 widowed.

### 2.2. Instruments

#### 2.2.1. Maslach Burnout Inventory Survey

The Maslach Burnout Inventory (MBI) is a tool aimed at assessing three main dimensions, including emotional exhaustion (EE), depersonalization (DP), and, finally, personal accomplishment (PA). It consists of 22 questions that are divided into three subscales (dimensions). The dimension EE (i.e., exhausted emotionally because of work) is measured by nine items (e.g., “I feel emotionally drained from my work”). DP concerns the impassive and impersonal response towards those receiving one’s service, care, treatment or instruction, loss of any positive attitude towards ourselves, the world, and others; it is measured by five items (e.g., “I feel I treat some friends as if they were impersonal objects”). PA is reduced personal competence, feelings of frustration, anger, loss of self-esteem, desire to change or leave the job, and lack of successful achievement in one’s work, and it is measured by eight items (e.g., “I feel I’m positively influencing other people’s lives through my work”). Each item is rated on a 7-point Likert-type scale ranging from 0 (never) to 6 (every day). Possible score ranges are 0 to 54 for EE (high risk: >26); moderate–high risk: 17–26; moderate or less: <17), 0 to 30 for DP (high risk: >12; moderate–high risk: 7–12; moderate or less: <7), and 0 to 48 for PA (high risk: <32; moderate–high risk: 32–38; moderate or less: >38). High BO levels are reflected in high scores on EE and DP and in low scores on PA. In our investigation, we assessed BO through the validated Italian-language version [18]. In the present study, internal consistency for the entire scale was α = 0.72. With respect to the subscale, EE α = 0.88, DP α = 0.75, and PA, α = 0.76. The results concord with earlier findings.

#### 2.2.2. Demographic, Education, and Job Characteristics

The questionnaire developed for this study asked the participants to indicate their gender, age, and education (e.g., parent’s role in career selection), job characteristics, and self-well-being (Table 2)

#### 2.2.3. The Toronto Alexithymia Scale

The Toronto Alexithymia Scale (TAS-20) is a 20-item self-report measure that assesses alexithymia, with each item rated on a five-point Likert scale. Total scores range from 20 to 100, with higher scores indicating higher alexithymia. The instrument includes three subscales: difficulty identifying feelings (DIF), difficulty describing feelings (DDF), and externally oriented thinking (EOT) or “concrete thinking style” [19]. The TAS-20 is a valid, widely used, and reliable measure of alexithymia. For the purposes of this study, we utilized the total score and subcomponent scores. Psychometric qualities of the Italian version of the TAS-20 were studied in a large clinical and nonclinical sample and can be considered adequate [20]. In the present study, internal consistency for the entire scale was α = 0.72. With respect to the subscale, EE α = 0.88, DP α = 0.75, and PA α = 0.76. The results are in line with previous findings.

#### 2.2.4. The Symptom Checklist-90-R

The Symptom Checklist-90-R (SCL-90-R) is a 90-item self-report inventory designed to reflect the psychological symptom patterns of psychiatric and medical patients. It is a measure of current (state) psychological symptom status [21]. The questionnaire is intended to measure the subjective self-reported severity of psychopathological symptoms. The SCL-90-R includes a number of different subscales exploring the severity of respondents’ symptoms over the previous seven days. Each item is rated on a 5-point Likert scale ranging from “Not at all” (0) to “Extremely” (4).

In clinical practice, the SCL-90-R is used to reflect an individual’s general symptom severity, as well as a more articulated subscale profile; the subscale profile is frequently adopted as an outcome measure in psychotherapy research [22].

The checklist consists of nine subscales and three global indexes of distress. The nine subscales are as follows: Somatization (SOM), Obsessive-Compulsive (O-C), Interpersonal sensitivity (I-S), Depression (DEP), Anxiety (ANX), Hostility (HOS), Phobic anxiety (PHOB), Paranoid ideation (PAR), and Psychoticism (PSY). The general indexes are as follows: Global Severity Index (GSI), Positive Symptom Total (PST), and Positive Symptom Distress Index (PSDI).

In particular:

- SOM reflects the discomfort linked to the perception of dysfunctions in one’s body; the symptoms focus on the cardiovascular, gastrointestinal, and respiratory systems. Pain and discomfort affecting gross muscles and other somatic equivalents of anxiety are also components of the scale.
- O-C includes symptoms characteristic of the clinical obsessive-compulsive syndrome; it focuses on thoughts, impulses, and actions experienced as persistent and irresistible, egodistonic, or unwanted in nature.
- I-S focuses on feelings of inadequacy and inferiority, especially in comparison to other people; characteristic manifestations are self-evaluation, self-doubt, and a marked discomfort in interpersonal interactions.
- DEP reflects a representative spectrum of clinical manifestations characteristic of the depressive syndrome; there is a withdrawal of interest in life, lack of motivation, and loss of vital energy; feelings of hopelessness, suicidal thoughts, and other cognitive and somatic correlates of depression are also included.
- ANX includes general signs of anxiety such as nervousness, tension, and tremors, as well as panic attacks and feelings of dread.
- HOS: reflects thoughts, feelings, and behaviors characteristic of a negative affective state of anger; it includes manifestations such as aggression, irritability, anger, and resentment.
- PHOB is defined as a persistent fear reaction to a specific person, place, object, or situation, perceived by the subject as irrational or disproportionate to the stimulus and which leads to avoidance or flight behavior.
- PAR describes the manifestations of paranoid thinking; projective thinking, hostility, suspiciousness, grandiosity, self-reference, fear of loss of autonomy, and delusions are all primary expressions of this disorder.
- PSY represents the construct as a continuous dimension of human experience and contains items indicative of withdrawal and isolation, as well as the first-rank symptoms of schizophrenia.

In the current study, we refer to the nine subscales and to the GSI as the single best indicator of the current level or depth of an individual’s disorder. It combines information concerning the number of symptoms reported with the intensity of perceived distress. The GSI is obtained by adding the scores of all 90 items and dividing by 90. In line with previous studies, in this investigation, Cronbach’s alpha for SCL90-GSI was 0.97 [23].

#### 2.2.5. Rosenberg Self-Esteem Scale

The global explicit self-esteem was measured by using the validated Italian version of the Rosenberg Self-Esteem Scale (RSES) [24]. The RSES consists of 10 closed questions measured on a Likert-like scale, where each item’s response ranges from 1 (strongly disagree) to 4 (strongly agree), and items are summed to produce a single index of self-esteem. The scale ranges from 10 to 40: five items positively and five negatively valence, these last reversely computed. While higher scores reflect a higher level of explicit self-esteem, we identified the cutoff of 30 as the threshold for high self-esteem [24]. Sample items included (“I feel that I have a number of good qualities”, “I am able to do things as well as most other people”). In the present study, internal consistency (Cronbach’s alpha), α = 0.86, concordant with earlier findings.

### 2.3. Data Analysis

To verify the hypothesis described above, we evaluated the associations between the study variables with different approaches and analyses that were taken using the SPSS 20 (IBM, 2017). Pearson’s correlations, analysis of variance, and linear regression models were used to analyze relationships among study variables. (i.e., age, sexual gender, and level of education).

Analyses were planned in four steps.

1. In the first step, we computed descriptive analysis of the demographic, education, job characteristics, and well-being questionnaire variables.
2. In the second step, we investigated the distribution of the MBI in the sample. To do that, we reported values above the high or moderate–high risk cutoffs for each component of the MBI test and determined the rates of individual forms.
3. In the third step, to examine the differences in the BO, alexithymia, symptoms, and self-esteem scores in terms of different categories defined in the general survey, we computed independent one-way ANOVAs.
4. Finally, the associations between variables were evaluated by examining Pearson’s correlations between the components of MBI (EE, DP, and PA scores), alexithymia global scores and components (DIF, DDF, and EOT), symptoms, and self-esteem. To investigate the quota of variance predicted in BO by alexithymia and symptoms dimensions, multiple independent linear regression analyses were performed

In the Results section (Section 3), categorical variables are shown as number and percentage (%). The significance level for all hypothesis testing was set at a *p*-value of minimum 0.05. Measures that had missing data were reported, and coefficients were reported standardized to facilitate interpretation.

## 3. Results

Descriptive analysis of the variables is reported in Table 3, in which the sociodemographic data of the sample are summarized. Burnout (BO) components were computed with standard cutoff scoring. With respect to the whole sample, 29% of individuals scored at high risk on emotional exhaustion (EE), followed by 36% at moderate–high risk. Depersonalization (DE) high and moderate–high risk were scored by 18.7% and 34.3% of individuals, respectively. Personal accomplishment (PA) with cutoff values of less than 38 points was scored by 34.7% of respondents. Results are reported in Table 4.

Rates of individual forms collected as both categorical and continuous variables were reported and analyzed by absolute and relative frequencies and by mean values and standard deviation (SD). One-way ANOVA results reported that significant differences in sexual gender were found in EE, where females scored significantly higher than males (male mean = 19.21; SD = 9.99; female mean = 22.17; SD = 10.27; F(1299) = 5.65; *p* < 0.001)

The highest mean scores of BO dimensions were related to dissatisfaction with one’s career, the feeling of being mobilized, conflicting relationship with colleagues and surgeons, and, finally, difficulty in explaining one’s work to patients (Table 5)

The total mean scores of job BO were significantly different among the participants of different sex and age groups (Table 5)

Associations between variables were evaluated by examining Pearson’s correlations between the components of MBI (EE, DP, and PA scores), alexithymia global scores and components (DIF, DDF, and EOT), symptoms, and self-esteem. As expected, most of the associations between study variables were significant. The highest and most significant were reported and discussed. All results are reported in Table 6.

The score of MBI-EE had the highest significant positive correlations with the Alexithymia DIF subscale (r = 0.52) and with symptoms: Obsessive-Compulsive (r = 0.51), Depression (r = 0.51), followed by interpersonal sensitivity (r = 0.47) and Anxiety (r = 0.44). The score of MBI DP was significantly positively associated only with Alexithymia (r = 0.26), whereas the highest association with symptoms was related to Interpersonal Sensitivity (r = 0.38). MBI-PA was significantly negatively associated with the EOT component of alexithymia (r = −0.20) and reported the highest negative association with Obsessive-Compulsive symptoms (r = −0.29).

Self-esteem was significantly negatively associated with the MBI-EE component (r = −0.31) to a greater extent than the other BO components.

To evaluate the extent to which alexithymia global scores and subscales (DIF, DDF, and EOT), global symptoms and subscales, and self-esteem predict their relationship with BO and MBI, component scores (EE, DE, PA) were examined as a criterion in a series of independent linear regression analyses. While collinearity was anticipated among subscales of the predictors, determining the association of alexithymia, symptoms, and self-esteem with BO is important in understanding and evaluating their unique and distinctive relation between them. More specifically, if a unique variance is predicted in BO by each component of alexithymia and symptoms dimensions, it would help determine how much overlap exists with these extant constructs and whether they have an explanation to support clinical Interpretations. To do that, multiple independent linear regression analyses were performed to predict the mean score of BO dimensions based on alexithymia, symptoms, and self-esteem. All analyses were eventually controlled for significant demographic characteristics (including age and gender). The regression models implemented in this section were defined to assess whether alexithymia, symptoms, and self-esteem components uniquely predict variance in BO component scores. The predictor variables of the regression analyses were alexithymia global score (TAS20 GS) and subscale scores (DIF, DDF, and EOT). Successively, a second set of analyses investigated symptom components in the prediction of the variance in BO component scores. The predictor variable of the first regression analysis was the symptoms’ global severity index (SCL-90-r GSI). In the second set of the analysis, all symptom subscales were entered simultaneously in the regression equation to evaluate the individual contribution to each BO component. Finally, in the last set of analyses, the global score of the self-esteem construct was implemented as a predictor variable of BO components in a series of independent regression analyses. All results are presented in Table 7.

As expected, alexithymia was significantly associated with all BO components. Alexithymia’s global score significantly best predicted MBI-EE component scores (β = 0.28), followed by a negatively significant association with MBI-PA (β = −0.23) and a positive significant association with MBI-DE (β = 0.19). With respect to alexithymia subscales, the DIF dimension was the unique significant predictor of all BO component score variance. In particular, DIF showed the highest positive significant association with MBI-EE (β = 0.33), followed by a positive significant association with MBI-DE (β = 0.27) and a small negative but significant association with MBI-PA (β = −0.11). Moreover, alexithymia’s EOT component showed a negative significant association with MBI-PA (β = −0.15).

With respect to psychological distress, BO’s MBI-EE component was significantly positively best predicted by Depression (β = 0.29), significantly negatively predicted by Psychoticism (β = −0.22), and significantly positively predicted by Obsessive-Compulsive (β = 0.21). Burnout’s MBI-DE component was significantly negatively predicted only by somatization (β = −0.23). Finally, BO’s MBI-PA component was significantly negatively best predicted by Obsessive-Compulsive (β = −0.25), followed by a negative significant association with Depression (β = −0.24).

As expected, associations between self-esteem construct and BO resulted in a positive significant association with the MBI-PA component (β = 0.25) and in a negative significant association with MBI-EE (β = −0.31), followed by a negative significant association with MBI-DE (β = −0.26).

## 4. Discussion

The COVID-19 pandemic has stressed health systems and health workers in an unprecedented way, inevitably causing a worsening of working conditions, with possible serious repercussions on the mental health conditions of workers [25,26]. However, the situation before the pandemic was not an optimal situation [10,27]. Precisely in this perspective, our study assumes remarkable importance. First of all, it presents itself as a baseline on which the burnout (BO) load due to the pandemic is grafted. Secondly, understanding what are the elements that influence BO in a nonemergency situation allows us to develop strategies capable of preventing and limiting this phenomenon when the epidemic situation has completely disappeared. The data in our possession confirm some elements established by the literature and common sense, such as high levels of BO and related symptoms in those who are not satisfied with their career [28]. However, new elements emerge that must lead us to reflect and to take measures. In fact, unlike other studies, we did not limit the study to evaluating the prevalence of BO and the association with psychophysical well-being (such as alcohol consumption), but we investigated the possible causes of BO related to the specificity of the profession of anesthesiologist/intensivist [6,9,10,11,12,13]. This difference is functional to understanding which elements can be modified through preventive health policies. The first major new element is the frustration that arises in having to explain one’s work to patients. The difficulty that patients and citizens in general encounter in understanding the indeed fundamental task carried out by intensivists is also evidenced by the media campaign during the pandemic that labeled them as heroes. In reality, there is nothing heroic in what has been done, but simply, professionalism played a key role in hospitals before [29,30]. Anesthesiologists complain of major difficulty in getting patients to understand their tasks, which is associated with significant scores in all three dimensions of BO, high scores for alexithymia, psychological symptoms of distress, and reduced self-esteem. This, in nonemergency conditions, is even more evident if we think of the fact that anesthesiologists recognize a greater burden on long and alienating shifts rather than on the suffering of patients and death. In general, these doctors are mentally prepared for a difficult and demanding job but suffer from shifts that do not allow them an acceptable quality of life (leisure capacity, quality of sleep) and conflict in the workplace [31]. In fact, even the conflict, they are not with or surgeons, but also and above all with their colleagues and their superiors is strongly associated with BO, alexithymia, and psychic symptoms. The percentage of subjects who would not undertake medical studies again is worrying (about 1/6), showing significant values of BO in all dimensions, associated with psychic symptoms. If we question such a strong and demanding choice as that of studying medicine, which, as Harrison wrote in the preface of his very famous treatise on internal medicine, is one of the most significant experiences in the life of a human being, it means that the psychological distress is unbearable [32]. Interventions are therefore needed at different levels. The first and most immediate is to use the right resources: economic, human, and technological. In fact, it is only by using resources that it is possible to reduce work shifts and make work less alienating. However, it is also necessary to change health policy, both in terms of recognition of the professional figure of the anesthesiologists and career prospects. By their nature, anesthetists are devoted to sacrifice and harsh self-criticism, and therefore, it is necessary to gratify them with the possibility of a career that enhances their skills [7,31]. In fact, our sample is dissatisfied with both career and salary. This, for example, is an element that strongly characterizes the Italian reality, in contrast to that of the US [6]. Last but not the least, think about systematic psychological interventions. Our data suggest that those who found themselves struggling with BO turned to psychotherapeutic support. These data should be read in a positive light; that is, these doctors are able to recognize the problem and ask for help when it becomes too big to be addressed alone.

## 5. Limitations

This study has some limitations that should be addressed by future research. First, it relies solely on self-report data, which are subject to errors in measurement. Considering the attitudes of people with high levels of personality features, it is possible that they tend to overestimate their psychological abilities and their own social functioning. Research strategies that combine the use of self-reports with external evaluations could possibly yield more accurate definitions of the relationship between “emotional” mental abilities and the different aspects of general personality. Second, a cross-sectional design was adopted which precludes causal inferences. Third, a sample of adults drawn from a health care professionals (HCPs) population was assessed, which does not allow researchers to evaluate the results within a more general population; this limit also concerns the potential association with not investigated personality characteristics and features. Directions for future research include ascertaining further the relative contributions of other personality features in association with symptoms or other clinically relevant variables. Finally, although the sample collected allowed us to carry out appropriate statistical analyzes, we failed to achieve the goal of recruiting a larger number of subjects. Because AAROI-EMAC (Italian Association of Hospital Anesthesiologists, Pain Medicine Specialists, Critical Care and Emergency Physicians) has approximately 10,000 members, the study was planned to enroll at least 1000 participants, corresponding to 10% of the members [17]. However, enrollment was stopped due to the COVID-19 pandemic. Including anesthesiologists engaged in the fight against the virus would have been logistically difficult and, above all, would have made the sample heterogeneous; it is obvious that the working conditions required by the pandemic such as extended work shifts, increased stress, etc., and the context itself of the unexpected emergency, may favor the triggering of the BO. Consequently, the sample would have been heterogeneous. On the other hand, we believe that these limitations may be a strength of our investigation. In particular, as it is a picture collected immediately before the COVID-19 crisis, it can be useful for evaluating the potentially traumatizing effects of the pandemic in this particular HCP setting.

## 6. Conclusions

In conclusion, among anesthesiologists, alexithymia components and symptom distress would explain an important portion of BO risk. The significant relationship between personality features and demographic and job characteristics may provide an important indication for clinically relevant risk factors indicators of BO in anesthesiologists and in their interpersonal and social functioning.

It is necessary to implement health policies in which support is provided for a category so at risk and that is at the same time of vital importance for the hospital economy [33]. Scientific societies, as indeed they are doing after the pandemic, can and must promote awareness campaigns on the role and importance of the anesthetist, to mitigate the knowledge gap that still exists among patients.

## Figures and Tables

**Table 1:** Inclusion and exclusion criteria.

Inclusion Criteria	Exclusion Criteria
Physicians with residency in anesthesia	Physicians with a history of psychiatric diagnoses
	Physicians with substance-related disorders
	Those with a history of psychotropic drugs

**Table 2:** Demographic, education, job characteristics, and well-being questionnaire.

Demographic data	
	Age
	Gender
	Have you a partner (Yes/No)
Education	
	How much yours parents have influenced your choice to study medicine? (Not at all; A little; A lot; They were against it)
	Going back in time, would you study medicine again? (Yes/No)
	Have you attended the course of specialization in the same University where you obtained your medical degree? (Yes/No)
	Was the speciality degree in anesthesiology your first choice? (Yes/No)
Job	
	In which Italian geographical area do you work? (North, Center, South, Islands)
	Do you work in the same hospital you have obtained the speciality degree? (Yes/No)
	Do you also carry out territorial emergency activities? (Yes/No)
	Do you also carry out helicopter rescue activities? (Yes/No)
	Is it your salary adequate to your expectations? (Yes/No)
	Are you satisfied with the activity you carry out? (Yes/No)
	Do you feel satisfied about your career? (Yes/No)
	Have you never received a complaint for professional reasons? (Yes/No)
Well-being	
	Do you have enough free time for your loved ones? (Yes/No)
	Do you have enough free time and energy to pursue hobbies? (Yes/No)
	Do you have enough free time and energy for sports? (Yes/No)
	Have you ever had the feeling of being mobbed? (Yes/No)
	Do you feel satisfied about your sleep quality? (Yes/No)
	Do you use benzodiazepines to promote your sleep? (Yes/No)
	Do you think the shift work is too stressful? (Yes/No)
	Does the lack of continuous contact with the patient create problems for you? (Yes/No)
	Have you a conflictual relationship with surgeons? (Low, High)
	Have you a conflictual relationship with other anesthesiologists? (A little, Very much)
	If you work in the operating room, is it a problem for you the light deprivation? (A little, Very much)
	Is it a problem for you to wear the operating room uniform? (A little, Very much)
	Is it hard for you to explain your work to patients? (Low, High)
	Do you think you are little considered by patients? (A little, Very much)
	Is the clinical severity of the patients you treat a problem for you? (A little, Very much)
	Have you ever benefited from psychotherapeutic? (Yes/No)

**Table 3:** Descriptive analysis of the variables.

Sex (n = 300)	n	%
Male	101	33.66
Female	199	66.33
Healthcare service type (n = 299)		
Private hospital	9	3.01
Public hospital	214	
IRCCS	21	71.57
University	23	7.023
Mixed hospital (private and public)	27	7.69
Ambulance	5	9.03
Missing	1	1.67
Geographical localization (n = 299)		
Northern Italy	160	53.51
Central Italy	78	26.08
Southern Italy	44	14.71
Islands	17	5.68
Missing	1	
Free professional contract	25	8.41
Fixed-term contract	37	12.45
Permanent contract	235	79.12
Missing	3	
Work organized on shifts	264	88.00
Not	36	12.00
I have a partner	265	88.33
I have not a partner	35	11.66
Civil Status		
Single	81	27.0
Married	196	65.3
Separate	11	3.7
Divorced	11	3.7
Widower	1	0.3

**Table 4:** Results of the Maslach Burnout Inventory.

MBI Factor	(Mean ± s.d.)	Median Score IQR	MBI Cutoff	Freq.	Freq. %
Emotional exhaustion	21.18 ± 10.25	14.00 (1–54)	High risk (>26)	88	29.3
			Moderate–high risk (17–26)	109	36.3
			Moderate or less (<17)	103	34.3
Depersonalization	7.04 ± 5.88	8.00 (0–24)	High risk (>12)	56	18.7
			Moderate–high risk (7–12)	87	29.0
			Moderate or less (<7)	157	52.3
Personal accomplishment	34.68 ± 7.31	11.00 (13–48)	High risk (<32)	94	31.3
			Moderate–high risk (32–38)	102	34.0
			Moderate or less (>38)	104	34.7

**Table 5:** Participants’ characteristics and total mean scores of Burnout and alexithymia: professional and private-life characteristics of respondents.

	Variables	f (f%)	MBI Emotional Exhaustion (EE) Mean ± SD; (F; *p*)	MBI Depersonalization (DE) Mean ± SD; (F; *p*)	MBI Personal Accomplishment (PA) Mean ± SD; (F; *p*)	TAS20 Alexithymia Mean ± SD; (F; *p*)	SCL-90-R Global Symptoms Mean ± SD; (F; *p*)	Self-Esteem Mean ± SD; (F; *p*)
Sex n = 300	Male	101 (33.7)	19.21 ± 9.99	7.68 ± 6.10	35.54 ± 7.19	42.96 ± 8.14	0.49 ± 0.38	23.70
	Female	199 (66.3)	22.17 ± 10.2 (5.65; 0.018 *)	6.71 ± 5.72 (1.82; n.s.)	34.24 ± 7.35 (2.11; n.s.)	43.93 ± 9.17 (0.00; n.s.)	0.58 ± 0.38 (3.03; n.s.)	22.52 (4.01; 0.04 *)
Age n = 300	25–39	117 (39.0)	20.17 ± 9.63	8.50 ± 6.03	34.48 ± 6.85	42.81 ± 9.10	0.57 ± 0.43	22.93 ± 4.80
	40–70	183 (64.0) (0.177, n.s.)	21.81 ± 10.6 (12.30; 0.001 *)	6.10 ± 5.61 (0.137; n.s.)	34.80 ± 7.61 (0.44; n.s.)	43.03 ± 8.67 (0.358; n.s.)	0.54 ± 0.35 (0.358; n.s.)	22.91 ± 4.84 (0.001, n.s.)
Would you study medicine again? n = 300	No	49 (16.33)	26.48 ± 11.5	9.57 ± 7.22	30.20 ± 7.83	44.28 ± 9.41	0.72 ± 0.51	20.79 ± 5.63
	Yes	251 (83.66)	20.14 ± 9.68 (16.50; 0.000 **)	6.54 ± 5.47 (11.19; 0.001 *)	35.55 ± 6.89 (23.61; 0.000 **)	42.68 ± 8.70 (1.34; n.s.)	0.52 ± 0.34 (11.56; 0.001 *)	23.33 ± 4.54 (11.80; 0.001 *)
Is it your salary adequate to your expectations? n = 296	No	231 (78.04)	22.26 ± 10.02	7.16 ± 5.93	34.61 ± 7.42	42.98 ± 8.95	0.55 ± 0.37	22.90 ± 4.70
	Yes	65 (21.95)	17.38 ± 10.30 (11.88; 0.001 *)	6.44 ± 5.59 (0.76; n.s.)	34.98 ± 6.85 (0.13; n.s.)	42.67 ± 8.19 (0.61; n.s.)	0.55 ± 0.42 (0.04; n.s.)	22.86 ± 5.34 (0.04; n.s.)
How afraid are you of getting a professional complaint? n = 297	A little Very Much	65 (21.88) 232 (78.11)	18.92 ± 10.83 21.90 ± 10.03 (4.32; 0.035 *)	5.50 ± 5.19 7.48 ± 5.99 (5.85; 0.016 *)	35.86 ± 7.87 34.31 ± 7.15 (2.28; n.s.)	42.12 ± 9.13 43.16 ± 8.74 (0.70; n.s.)	0.44 ± 0.28 0.58 ± 0.40 (6.542; 0.011 *)	23.56 ± 5.12 22.78 ± 4.70 (1.344; n.s.)
Are you satisfied with the activity you carry out? n = 298	No	101 (33.89)	25.84 ± 10.28	8.84 ± 6.05	32.75 ± 7.19	43.34 ± 9.46	0.62 ± 0.39	21.51 ± 5.17
	Yes	197 (66.10)	18.78 ± 9.46 (34.96; 0.000 **)	6.17 ± 5.60 (14.32; 0.000** )	35.69 ± 7.22 (11.06; 0.001 *)	42.62 ± 8.44 (0.46; n.s.)	0.51 ± 0.37 (5.75; 0.017 *)	23.69 ± 4.47 (14.16; 0.000 **)
Do you have enough free time and energy for sports? n = 299	No	134 (44.81)	23.61 ± 10.37	7.04 ± 5.83	34.05 ± 7.43	43.82 ± 9.57	0.60 ± 0.42	22.51 ± 5.10
	Yes	165 (55.1)	19.23 ± 9.78 (14.04; 0.000 *)	7.05 ± 5.96 (0.00; n.s.)	35.15 ± 7.21 (1.66; n.s.)	42.24 ± 8.15 (2.37; n.s.)	0.51 ± 0.35 (3.73; n.s.)	23.27 ± 4.57 (1.85; n.s.)
Have you ever had the feeling of being mobbed? n = 300	No	167 (55.6)	19.01 ± 10.37	6.67 ± 5.49	35.04 ± 7.30	43.08 ± 8.77	0.49 ± 0.34	22.94 ± 4.84
	Yes	133(44.33)	23.90 ± 9.46 (17.77; 0.000 **)	7.49 ± 6.34 (1.14; n.s.)	34.23 ± 7.33 (0.95; n.s.)	42.77 ± 8.93 (0.91; n.s.)	0.62 ± 0.42 (9.04; 0.003 *)	22.90 ± 4.81 (0.05; n.s.)
If you feel mobilized, do you feel mobilized by a colleague or a superior? n = 133	Colleague	38 (28.57)	24.60 ± 10.07	8.68 ± 7.06	34.97 ± 7.13	42.05 ± 7.65	0.81 ± 0.51	22.23 ± 4.58
	Superior	95 (71.43)	23.62 ± 9.25 (0.29; n.s.)	7.02 ± 6.00 (1.87; n.s.)	33.93 ± 7.43 (0.54, n.s.)	43.06 ± 9.40 (0.34; n.s.)	0.55 ± 0.35 (11.38, 0.001 *)	23.16 ± 4.90 (1.01, n.s.)
Do you feel satisfied about your career? n= 300	No	139 (46.33)	24.15 ± 10.23	7.79 ± 5.82	33.35 ± 7.05	43.41 ± 9.03	0.63 ± 0.39	21.61 ± 4.76
	Yes	161 (53.66)	18.61 ± 9.59 (23.35; 0.000 **)	6.39 ± 5.88 (4.26; 0.040 *)	35.82 ± 7.36 (8.67; 0.03 *)	42.54 ± 8.65 (0.73; n.s.)	0.48 ± 0.37 (10.42; 0.001 *)	24.05 ± 4.59 (20.39; 0.000 **)
Do you feel satisfied about your sleep quality? n = 299	No	187 (62.54)	23.25 ± 10.11	7.45 ± 6.26	34.61 ± 7.22	42.96 ± 9.30	0.58 ± 0.38	22.47 ± 5.01
	Yes	112 (37.45)	17.76 ± 9.63 (21.33; 0.000 **)	6.38 ± 5.16 (2.34; n.s.)	34.78 ± 7.53 (0.038; n.s.)	42.98 ± 8.02 (0.00; n.s.)	0.51 ± 0.39 (2.00; n.s.)	23.64 ± 4.42 (4.17; 0.042 *)
Do you use benzodiazepines to promote your sleep? n = 299	No	277 (92.64)	20.77 ± 10.10	6.83 ± 5.73	35.01 ± 7.20	42.75 ± 8.78	0.54 ± 0.37	22.99 ± 4.73
	Yes	22 (7.35)	26.77 ± 10.72 (7.11; 0.008 *)	9.77 ± 7.28 (5.13; 0.024 *)	32.22 ± 7.39 (5.61; 0.018 *)	45.09 ± 9.43 (1.43; n.s.)	0.72 ± 0.47 (4.51; 0.034 *)	22.18 ± 5.98 (0.57; n.s.)
Do you think the shift work is too stressful? n = 298	No	104 (34.89)	18.74 ± 10.65	6.68 ± 5.91	34.59 ± 7.33	42.68 ± 9.48	0.53 ± 0.37	23.51 ± 4.68
	Yes	160 (53.69)	23.06 ± 9.63 (6.21, 0.002 *)	7.47 ± 5.88 (1.21; n.s.)	34.50 ± 6.97 (0.52; n.s.)	42.97 ± 8.58 (0.17; n.s.)	0.57 ± 0.39 (0.41; n.s.)	22.59 ± 4.73 (1.24; n.s.)
Have you a conflictual relationship with surgeons? n = 298	Low	182 (61.07)	19.62 ± 10.31	5.79 ± 5.53	35.31 ± 7.56	42.84 ± 9.12	0.50 ± 0.34	23.52 ± 4.76
	High	116 (38.92)	23.62 ± 9.79 (11.05, 0.001 *)	8.89 ± 5.77 (21.58; 0.000 **)	33.65 ± 6.86 (3.65; n.s.)	43.16 ± 8.43 (0.09; n.s.)	0.64 ± 0.42 (9.86; 0.002 *)	21.94 ± 4.79 (7.73; 0.006 *)
Have you a conflictual relationship with other anesthesiologists? n = 300	Low	126 (42.00)	19.53 ± 10.45	5.65 ± 5.00	35.12 ± 7.44	43.11 ± 9.22	0.51 ± 0.36	22.77 ± 5.33
	High	176 (56.00)	22.37 ± 9.97 (5.69, 0.018 *)	8.04 ± 6.27 (12.41; 0.000 **)	34.36 ± 7.22 (0.79; n.s.)	42.82 ± 8.55 (0.08; n.s.)	0.58 ± 0.39 (1.94; n.s.)	23.02 ± 4.43 (0.19; n.s.)
Is it hard for you to explain your work to patients? n = 300	Low	159 (53.00)	18.11 ± 9.57	5.42 ± 5.12	36.07 ± 7.31	41.72 ± 7.93	0.46 ± 0.29	23.74 ± 4.39
	High	141 (47.00)	24.63 ± 9.93 (33.37; 0.000 **)	8.86 ± 6.17 (27.85; 0.000 **)	33.11 ± 7.02 (12.73; 0.000 **)	44.31 ± 9.58 (6.54; 0.011 *)	0.65 ± 0.44 (17.44; 0.000 **)	22.00 ± 5.12 (10.03; 0.002 *)
Is the clinical severity of the patients you treat a problem for you? n = 298	Low	101 (33.89)	18.05 ± 10.51	6.58 ± 6.24	35.60 ± 7.56	41.90 ± 8.26	0.49 ± 0.40	23.37 ± 4.87
	High	197 (66.10)	22.81 ± 9.76 (15.01; 0.000 **)	7.22 ± 5.67 (0.79; n.s.)	34.22 ± 7.14 (2.37; n.s.)	43.38 ± 9.05 (1.93; n.s.)	0.58 ± 0.37 (3.26; n.s.)	22.70 ± 4.74 (1.32; n.s.)
What weighs you the most? n = 298	Death of a patient	85 (28.52)	21.12 ± 11.20	6.62 ± 5.74	34.37 ± 7.51	44.20 ± 8.58	0.57 ± 0.47	22.67 ± 4.89
	Suffering of a patient	155 (52.01)	20.57 ± 9.43	6.38 ± 5.41	35.31 ± 7.00	42.52 ± 8.78	0.55 ± 0.36	22.92 ± 4.66
	Long work shifts	58 (19.46)	22.93 ± 11.05 (1.11; n.s.)	9.62 ± 6.65 (7.01; 0.001 *)	33.18 ± 7.72 (1.86; n.s.)	42.22 ± 9.39 (1.21; n.s.)	0.52 ± 0.31 (0.36; n.s.)	23.20 ± 5.24 (0.21; n.s.)
Do you have enough free time for your loved ones? n = 300	No	206 (68.66)	31.17 ± 9.28	7.57 ± 6.17	34.37 ± 7.13	43.39 ± 9.08	0.58 ± 0.38	22.51 ± 4.81
	Yes	94 (31.33)	16.79 ± 10.95 (27.17; 0.000 **)	5.87 ± 5.05 (5.46; 0.020 *)	35.36 ± 7.68 (1.17; n.s.)	41.96 ± 8.21 (1.68; n.s.)	0.49 ± 0.39 (2.89; n.s.)	23.80 ± 4.75 (4.66; 0.032 *)
Have you ever benefited from psychotherapeutic? n = 300	No	233 (77.66)	20.02 ± 9.77	6.61 ± 5.65	34.68 ± 7.27	42.56 ± 8.46	0.48 ± 0.32	23.36 ± 4.74
	Yes	67 (22.33)	25.19 ± 10.94 (13.77; 0.000 **)	8.50 ± 6.47 (5.43; 0.020 *)	34.67 ± 7.49 (0.00; 0.020 *)	44.26 ± 9.95 (1.94; n.s.)	0.80 ± 0.48 (40.13; 0.000 **)	21.40 ± 4.81 (8.79; 0.003 *)

Legend. * *p* < 0.05; ** *p* < 0.001. n.s. = not significant

**Table 6:** Prediction of MBI subscales in Alexithymia, Symptoms Distress (SCL-90-R) Global Scores and Subscales, and Rosenberg Self-Esteem (RSE) (n = 300).

	MBI Emotional Exhaustion (EE) *Std. β (S.E)*	MBI Depersonalization (DE) *Std. β (S.E)*	MBI Personal Accomplishment (PA) *Std. β (S.E)*	Models Parameters R^2^; F; *p* MBI-EE ^a^; MBI-DE ^b^; MBI-PA ^c^
Predictor				
TAS20 G.S.	0.28 ** (0.06)	0.19 ** (0.03)	−0.23 ** (0.04)	^a^ 0.08; 25.54; 0.000 ^b^ 0.04; 12.19; 0.001 ^c^ 0.05; 16.77; 0.000
Difficulties in identifying	0.39 ** (0.13)	0.27 ** (0.08)	−0.11 ** (0.10)	^a^ 0.14; 16.39; 0.000
Difficulties describing feelings	−0.03 n.s. (0.25)	−0.00 n.s. (0.15)	−0.03 n.s. (0.19)	^b^ 0.07; 7.47; 0.000
External oriented thinking	−0.00 n.s. (0.14)	−0.01 n.s. (0.08)	−0.15 *. (0.10)	^c^ 0.05; 5.78; 0.001
SCL90 G.S.I.	0.52 ** (1.03)	0.33 ** (1.03)	−0.25 ** (1.06)	^a^ 0.27; 111.22; 0.000 ^b^ 0.13; 45.81; 0.001 ^c^ 0.06; 18.88; 0.000
Somatization	0.02 n.s. (0.12)	−0.23 * (0.07)	0.07 n.s. (0.10)	^a^ 0.31; 14.80; 0.000
Obsessive-Compulsive	0.21 * (0.16)	0.11 n.s. (0.10)	−0.25 * (0.13)	^b^ 0.19; 7.54; 0.000
Interpersonal Sensitivity	0.14 n.s. (0.22)	0.12 n.s. (0.14)	0.03 n.s. (0.18)	^c^ 0.10; 3.69; 0.001
Depression	0.29 * (0.16)	0.09 n.s. (0.10)	−0.24 * (0.13)	
Anxiety	0.03 n.s. (0.22)	0.11 n.s. (0.13)	0.05 n.s. (0.18)	
Hostility	0.08 n.s. (0.23)	0.09 n.s. (0.14)	0.01 n.s. (0.18)	
Phobic Anxiety	0.03 n.s. (0.34)	0.04 n.s. (0.21)	−0.05 n.s. (0.28)	
Paranoid Ideation	−0.02 n.s. (0.27)	0.11 n.s. (0.17)	0.07 n.s. (0.22)	
Psychoticism	−0.22 * (0.25)	−0.05 n.s. (0.16)	0.12 n.s. (0.20)	
R. Self-Esteem G.S.	−0.31 ** (0.11)	−0.26 ** (0.06)	0.25 ** (0.08)	^a^ 0.10; 32.90; 0.000 ^b^ 0.07; 22.55; 0.000 ^c^ 0.06; 21.67; 0.000

Legend. Criterion: Maslach Burnout Inventory, Emotional Exhaustion, Depersonalization, Personal Accomplishment. Predictors: Toronto Alexithymia Scale: Difficulties in Identifying Feelings (DIF), Difficulties Describing Feelings (DDF), External Oriented Thinking (EOT). Symptom Checklist Global Severity Index (SCL90R-GSI), Somatization, Obsessive-Compulsive, Interpersonal Sensitivity, Depression, Anxiety, Hostility, Phobic Anxiety, Paranoid Ideation, Psychoticism. Rosenberg Self-Esteem (RSE) * *p* < 0.05; ** *p* < 0.001. ^a^ = MBI-EE; ^b^ = MBI-DE; ^c^ = MBI-PA.

**Table 7:** Correlations between MBI subscales in Alexithymia and Symptoms Distress (SCL-90-R) Global Scores and Subscales, and Rosenberg Self-Esteem (RSE) (n = 300).

	MBI Emotional Exhaustion	MBI Depersonalization	MBI Personal Accomplishment	TAS20 G.S.	TAS20 Difficulties Identifying Feelings	TAS20 Difficulties Describing Feelings	TAS20 External Oriented Thinking	RSE Self-Esteem
TAS20 G.S.	0.281	0.198	−0.231 **	-				−0.452 **
TAS Difficulties in identifying	0.376 **	0.265 **	0.170 **	0.788 **				−0.459 **
TAS Difficulties Described feelings	0.135 *	0.109	−0.148 *	0.735 **	0.438 **			−0.300 **
TAS External oriented thinking	0.095	0.06	−0.203 **	0.768 **	0.289 **	0.439 **		−0.258 **
SCL90 G.S.I.	0.525 **	0.366 **	−0.252 **	0.466 **	0.544 **	0.256 **	0.219 **	−0.492 **
SCL90 Somatization	0.357 **	0.103	−0.138 *	0.377 **	0.455 **	0.188 **	0.174 **	−0.222 **
SCL90 Obsessive-Compulsive	0.510 **	0.330 **	−0.292 **	0.514 **	0.569 **	0.338 **	0.244 **	−0.550 **
SCL90 Interpersonal Sensitivity	0.472 **	0.380 **	−0.223 **	0.355 **	0.418 **	0.250 **	0.132*	−0.468 **
SCL90 Depression	0.513 **	0.355 **	−0.265 **	0.405 **	0.463 **	0.234 **	0.196 **	−0.553 **
SCL90 Anxiety	0.446 **	0.312 **	−0.197 **	0.357 **	0.459 **	0.146 *	0.156 **	−0.395 **
SCL90 Hostility	0.366 **	0.316 **	−0.144 *	0.351 **	0.400 **	0.177 **	0.188 **	−0.261 **
SCL90 Phobic Anxiety	0.302 **	0.228 **	−0.157 **	0.388 **	0.366 **	0.257**	0.252 **	−0.302 **
SCL90 Paranoid Ideation	0.379 **	0.358 **	−0.158 **	0.297 **	0.403 **	0.165 **	0.081	−0.324 **
SCL90 Psychoticism	0.328 **	0.309 **	−0.150 **	0.368 **	0.415 **	0.186 **	0.200 **	−0.363 **
RSE Self-Esteem	−0.315 **	−0.265 **	0.258 **					

Note: Criterion: Maslach Burnout Inventory, Emotional Exhaustion, Depersonalization, Personal Accomplishment. Predictors: Toronto Alexithymia Scale: Difficulties in Identifying Feelings (DIF), Difficulties Describing Feelings (DDF), External Oriented Thinking (EOT). Symptom Checklist: Global Severity Index (SCL90R-GSI), Somatization, Obsessive-Compulsive, Interpersonal Sensitivity, Depression, Anxiety, Hostility, Phobic Anxiety, Paranoid Ideation, Psychoticism. Rosenberg Self-Esteem (RSE) * *p* < 0.05; ** *p* < 0.001.

## Data Availability

The datasets used and/or analyzed during the current study are available from the corresponding author on reasonable request.
